# Supplementary material for: Factorized discriminant analysis for genetic signatures of neuronal phenotypes
Source: Front Neuroinform. 2023 Dec 14;17:1265079. doi: 10.3389/fninf.2023.1265079 (PMC10752939; doi:10.3389/fninf.2023.1265079)
Supplement: Supplementary file 1 [file Data_Sheet_1.pdf]

Factorized Discriminant Analysis for Genetic Signatures of Neuronal Phenotypes

Mu Qiao[1,2]
[1]Division of Biology and Biological Engineering,
California Institute of Technology, Pasadena, CA 91125
[2]Present address: LinkedIn, Mountain View, CA, 94043
muqiao0626@gmail.com

1 Appendix

1.1 A. Objective functions

Here we derive the objective functions used in our analysis. Again if $\boldsymbol{x}_{ijk}(k \in 1, 2, ...n_{ij})$ represents the expression values of g genes in each cell ($\boldsymbol{x}_{ijk} \in \mathbf{R}^g$)), we seek to find a linear projection $y_{ijk} = \boldsymbol{u}^T \boldsymbol{x}_{ijk}$ that is aligned with the feature i.

1.1.1 Inspiration from ANOVA

We asked what is the best way to factorize y_{ijk}. Inspired by multi-way ANOVA [1], we identified three components: one depending on the feature i, another depending on the feature j, and the last one depending on the combination of both features. We therefore followed the procedures of ANOVA to partition sums of squares and factorize y_{ijk} into these three components.

Let us first assume that all cell types defined by i and j contain the same number of cells. With cell types represented by a complete contingency table (Figure 1A), y_{ijk} can be linearly factorized using the model of two crossed factors. Formally, the linear factorization is the following:

$$y_{ijk} = \mu + \alpha_i + \beta_j + (\alpha\beta)_{ij} + \epsilon_{ijk} \tag{1}$$

where y_{ijk} represents the coordinate of the kth cell in the category defined by i and j; μ is the average level of y; α_i is the component that depends on the feature i, and β_j is the component that depends on the feature j; $(\alpha\beta)_{ij}$ describes the component that depends on the combination of both features i and j; $\epsilon_{ijk} \sim \mathcal{N}(0, \sigma^2)$ is the residual of this factorization.

Let us say that the features i and j fall into a and b discrete categories respectively. Then without loss of generality, we can require:

$$\sum_{i=1}^{a} \alpha_i = 0 \tag{2}$$

$$\sum_{j=1}^{b} \beta_j = 0 \tag{3}$$

$$\sum_{i=1}^{a} (\alpha\beta)_{ij} = \sum_{j=1}^{b} (\alpha\beta)_{ij} = 0 \tag{4}$$

Preprint.

Corresponding to these, there are three null hypotheses:

$$H_{01} : \alpha_i = 0 \tag{5}$$

$$H_{02} : \beta_j = 0 \tag{6}$$

$$H_{03} : (\alpha\beta)_{ij} = 0 \tag{7}$$

Here we want to reject H_{01} while accepting H_{02} and H_{03} so that y_{ijk} is aligned with the feature i.

Next, we partition the total sum of squares. If the number of cells within each cell type category is n, and the total number of cells is N, then we have

$$\begin{aligned}\sum_{i=1}^{a}\sum_{j=1}^{b}\sum_{k=1}^{n}(y_{ijk} - \bar{y}_{...})^2 = bn\sum_{i=1}^{a}(\bar{y}_{i..} - \bar{y}_{...})^2 + an\sum_{j=1}^{b}(\bar{y}_{.j.} - \bar{y}_{...})^2 \\ + n\sum_{i=1}^{a}\sum_{j=1}^{b}(\bar{y}_{ij.} - \bar{y}_{i..} - \bar{y}_{.j.} + \bar{y}_{...})^2 + \sum_{i=1}^{a}\sum_{j=1}^{b}\sum_{k=1}^{n}(y_{ijk} - \bar{y}_{ij.})^2\end{aligned} \tag{8}$$

where $\bar{y}$ is the average of y_{ijk} over the indices indicated by the dots. Equation (8) can be written as

$$SS_T = SS_A + SS_B + SS_{AB} + SS_e \tag{9}$$

with each term having degrees of freedom $N-1$, $a-1$, $b-1$, $(a-1)(b-1)$, and $N-ab$ respectively. Here SS_A, SS_B, SS_{AB}, and SS_e are partitioned sum of squares for the factors α_i, β_j, $(\alpha\beta)_{ij}$, and the residual.

ANOVA rejects or accepts a null hypothesis by comparing its mean square (the partitioned sum of squares normalized by the degree of freedom) to that of the residual. This is done by constructing F-statistics for each factor as shown below:

$$F_A = \frac{MS_A}{MS_e} = \frac{\frac{SS_A}{a-1}}{\frac{SS_e}{N-ab}} \tag{10}$$

$$F_B = \frac{MS_B}{MS_e} = \frac{\frac{SS_B}{b-1}}{\frac{SS_e}{N-ab}} \tag{11}$$

$$F_{AB} = \frac{MS_{AB}}{MS_e} = \frac{\frac{SS_{AB}}{(a-1)(b-1)}}{\frac{SS_e}{N-ab}} \tag{12}$$

Under the null hypotheses, the F-statistics follow the F-distribution. Therefore, a null hypothesis is rejected when we observe the value of a F-statistic above a certain threshold calculated from the F-distribution. Here we want F_A to be large enough so that we can reject H_{01}, but F_B and F_{AB} to be small enough for us to accept H_{02} and H_{03}. In other words, we want to maximize F_A while minimizing F_B and F_{AB}. Therefore, we propose maximizing an objective L:

$$L = F_A - \lambda_1 F_B - \lambda_2 F_{AB} \tag{13}$$

where λ_1 and λ_2 are hyper-parameters determining the relative weights of F_B and F_{AB} compared with F_A.

1.1.2 Objective functions under a complete contingency table

When the numbers of cells within categories defined by i and j (n_{ij}) are not all the same, the total sum of squares cannot be partitioned as in Equation (8). However, if we only care about distinctions between cell types instead of individual cells, we can use the mean value of each cell type cluster ($\bar{y}_{ij.}$) to estimate the overall average value ($\tilde{y}_{...}$), and the average value of each category i ($\tilde{y}_{i..}$) or j ($\tilde{y}_{.j.}$). Therefore, Equation (8) can be modified as the following:

$$\sum_{i=1}^{a}\sum_{j=1}^{b}[\frac{1}{n_{ij}}\sum_{k=1}^{n_{ij}}(y_{ijk}-\tilde{y}_{...})^2] = b\sum_{i=1}^{a}(\tilde{y}_{i..}-\tilde{y}_{...})^2 + a\sum_{j=1}^{b}(\tilde{y}_{.j.}-\tilde{y}_{...})^2 + \sum_{i=1}^{a}\sum_{j=1}^{b}(\bar{y}_{ij.}-\tilde{y}_{i..}-\tilde{y}_{.j.}+\tilde{y}_{...})^2 + \sum_{i=1}^{a}\sum_{j=1}^{b}[\frac{1}{n_{ij}}\sum_{k=1}^{n_{ij}}(y_{ijk}-\bar{y}_{ij.})^2] \tag{14}$$

where

$$\bar{y}_{ij.} = \frac{\sum_{k=1}^{n_{ij}} y_{ijk}}{n_{ij}} \tag{15}$$

$$\tilde{y}_{i..} = \frac{\sum_{j=1}^{b} \bar{y}_{ij.}}{b} \tag{16}$$

$$\tilde{y}_{.j.} = \frac{\sum_{i=1}^{a} \bar{y}_{ij.}}{a} \tag{17}$$

$$\tilde{y}_{...} = \frac{\sum_{i=1}^{a}\sum_{j=1}^{b} \bar{y}_{ij.}}{ab} \tag{18}$$

If we describe Equation (14) as:

$$\tilde{SS}_T = \tilde{SS}_A + \tilde{SS}_B + \tilde{SS}_{AB} + \tilde{SS}_e \tag{19}$$

then following the same arguments, we want to maximize an objective function in the following format:

$$L = \frac{\frac{\tilde{SS}_A}{a-1} - \lambda_1\frac{\tilde{SS}_B}{b-1} - \lambda_2\frac{\tilde{SS}_{AB}}{(a-1)(b-1)}}{\frac{\tilde{SS}_e}{N-ab}} \tag{20}$$

1.1.3 Objective functions under a partial contingency table

When we have a representation of a partial table, we can no longer separate out the component that depends on the combination of both features. Therefore, we use another model, a linear model of two nested factors, to factorize y_{ijk}, which has the following format:

$$y_{ijk} = \mu + \alpha_i + \beta_{j(i)} + \epsilon_{ijk} \tag{21}$$

Note that we now have $\beta_{j(i)}$ instead of $\beta_j + (\alpha\beta)_{ij}$. In this model, we identify a primary factor, for instance, the feature denoted by i which falls into a categories, and the other (indexed by j) becomes a secondary factor, the number of whose levels b_i depends on the level of the primary factor. We merge the component depending on the combination of both features into that of the secondary factor as $\beta_{j(i)}$.

Similarly, we have

$$\sum_{i=1}^{a}\sum_{j=1}^{b_i}[\frac{1}{n_{ij}}\sum_{k=1}^{n_{ij}}(y_{ijk}-\tilde{y}_{...})^2] = \sum_{i=1}^{a}[\sum_{j=1}^{b_i}(\tilde{y}_{i..}-\tilde{y}_{...})^2] + \sum_{i=1}^{a}\sum_{j=1}^{b_i}(\bar{y}_{ij.}-\tilde{y}_{i..})^2 + \sum_{i=1}^{a}\sum_{j=1}^{b_i}[\frac{1}{n_{ij}}\sum_{k=1}^{n_{ij}}(y_{ijk}-\bar{y}_{ij.})^2] \tag{22}$$

which can be written as

$$\tilde{SS}_T = \tilde{SS}_A + \tilde{SS}_B + \tilde{SS}_e \tag{23}$$

with degrees of freedom $N-1$, $a-1$, $M-a$, and $N-M$ for each of the terms, where M is:

$$M = \sum_{i=1}^{a} b_i \tag{24}$$

Therefore, we want to maximize the following objective:

$$L = \frac{\frac{\tilde{SS}_A}{a-1} - \lambda\frac{\tilde{SS}_B}{M-a}}{\frac{\tilde{SS}_e}{N-M}} \tag{25}$$

1.2 B. FLDA with a partial contingency table

Here we provide the mathematical details of FLDA under the representation of a partial table. When we have a partial table, if the feature i is the primary feature with a levels, and the feature j is the secondary feature with b_i levels, then $\boldsymbol{N}_A$ is defined as follows:

$$\boldsymbol{N}_A = \boldsymbol{M}_A - \lambda\boldsymbol{M}_{B|A} \tag{26}$$

where

$$\boldsymbol{M}_A = \frac{1}{a-1}\sum_{i=1}^{a}\sum_{j=1}^{b_i}(\boldsymbol{m}_{i.}-\boldsymbol{m}_{..})(\boldsymbol{m}_{i.}-\boldsymbol{m}_{..})^T \tag{27}$$

$$\boldsymbol{M}_{B|A} = \frac{1}{M-a}\sum_{i=1}^{a}\sum_{j=1}^{b_i}(\boldsymbol{m}_{ij}-\boldsymbol{m}_{i.})(\boldsymbol{m}_{ij}-\boldsymbol{m}_{i.})^T \tag{28}$$

and M is defined as in Equation (24). Correspondingly, $\boldsymbol{M}_e$ is defined as:

$$\boldsymbol{M}_e = \frac{1}{N-M}\sum_{i=1}^{a}\sum_{j=1}^{b}[\frac{1}{n_{ij}}\sum_{k=1}^{n_{ij}}(\boldsymbol{x}_{ijk}-\boldsymbol{m}_{ij})(\boldsymbol{x}_{ijk}-\boldsymbol{m}_{ij})^T] \tag{29}$$

and

$$\boldsymbol{m}_{..} = \frac{1}{M}\sum_{i=1}^{a}\sum_{j=1}^{b_i}\boldsymbol{m}_{ij} \tag{30}$$

$$\boldsymbol{m}_{i.} = \frac{1}{b_i}\sum_{j=1}^{b_i}\boldsymbol{m}_{ij} \tag{31}$$

The remaining mathematical arguments are the same as those for the complete table. In this scenario, because we don't observe all possible combinations of features i and j, we cannot find the linear projection for the combination of both features.

1.3 C. Implementation details of data synthesis

To quantitatively compare FLDA with alternative approaches, we synthesized data of four cell types, each of which contained 250 cells. The four cell types were generated from a Cartesian product of two features i and j, where $i \in \{0, 1\}$ and $j \in \{0, 1\}$. Expressions of 1000 genes were generated for each cell. The expression value of the hth gene in the kth cell of the cell type ij, x^{h}_{ijk} was defined as the following:

$$x^{1:100}_{ijk} = i + \epsilon_{ijk} \tag{32}$$

$$x^{101:200}_{ijk} = j + \epsilon_{ijk} \tag{33}$$

$$x^{201:300}_{ijk} = i \wedge j + \epsilon_{ijk} \tag{34}$$

$$x^{301:400}_{ijk} = i \vee j + \epsilon_{ijk} \tag{35}$$

$$x^{401:500}_{ijk} = 2i + \epsilon_{ijk} \tag{36}$$

$$x^{501:600}_{ijk} = 2j + \epsilon_{ijk} \tag{37}$$

$$x^{601:700}_{ijk} = 2i \wedge j + \epsilon_{ijk} \tag{38}$$

$$x^{701:800}_{ijk} = 2i \vee j + \epsilon_{ijk} \tag{39}$$

$$x^{801:900}_{ijk} = \epsilon_{ijk} \tag{40}$$

$$x^{901:1000}_{ijk} = 2 + \epsilon_{ijk} \tag{41}$$

where

$$i \wedge j = \begin{cases} 1, & \text{if } i = 1, j = 1 \\ 0, & \text{otherwise} \end{cases} \tag{42}$$

and

$$i \vee j = \begin{cases} 0, & \text{if } i = 0, j = 0 \\ 1, & \text{otherwise} \end{cases} \tag{43}$$

were combinations of the two features. Here ϵ_{ijk} represents Gaussian noise, namely

$$\epsilon_{ijk} \sim \mathcal{N}(0, \sigma^2) \tag{44}$$

We generated synthetic datasets under varying levels of Gaussian noise by adjusting the σ value across a set of 5 distinct levels ($\sigma \in 2, 4, 6, 8, 10$). For each of these σ values, we created 10 distinct datasets in a repetitive manner, leading to a total of 50 synthetic datasets. The evaluation metrics for each σ value were then computed as the average across the respective 10 repetitions, providing a robust evaluation of the proposed FLDA method across different noise levels.

1.4 D. Implementation details of the metrics used in the study

We measured the following metrics in our experiments:

1.4.1 Signal-to-Noise Ratio (SNR)

Because we care about the separation of cell types, we define the SNR metric as the ratio of the variance between cell types over the variance of the noise, which is estimated from within-cluster variance. For the entire embedding space, given q cell types, if the coordinate of each cell is indicated by $\boldsymbol{x}$, then we define the overall SNR metric as the following:

$$SNR_{overall} = \frac{tr(\Sigma_{p=1}^{q} n_p(\bar{\boldsymbol{x}}_{p.} - \bar{\boldsymbol{x}}_{..})(\bar{\boldsymbol{x}}_{p.} - \bar{\boldsymbol{x}}_{..})^T))}{tr(\Sigma_{p=1}^{q} \Sigma_{k=1}^{n_p}(\boldsymbol{x}_{pk} - \bar{\boldsymbol{x}}_{p.})(\boldsymbol{x}_{pk} - \bar{\boldsymbol{x}}_{p.})^T)} \tag{45}$$

where $\bar{\boldsymbol{x}}_{p.}$ is the center of each cell type cluster, and $\bar{\boldsymbol{x}}_{..}$ is the center of all data points.

Let x denote the embedded coordinate along a specific dimension. The SNR metric for that axis is therefore:

$$SNR = \frac{\Sigma_{p=1}^{q} n_p(\bar{x}_{p.} - \bar{x}_{..})^2}{\Sigma_{p=1}^{q} \Sigma_{k=1}^{n_p}(x_{pk} - \bar{x}_{p.})^2} \tag{46}$$

1.4.2 Explained Variance (EV)

We want to know whether the variation of a specific dimension is strongly explained by that of a specific feature. Therefore, we measure, for each axis, how much of the total explained variance is explained by the variance of the feature i or j. Formally, given the embedded coordinate x_{ijk}, we calculate the EV as the following:

$$EV_i = \frac{\sum_{i=1}^{a} \sum_{j=1}^{b} n_{ij}(\bar{x}_{i..} - \bar{x}_{...})^2}{\sum_{i=1}^{a} \sum_{j=1}^{b} \sum_{k=1}^{n_{ij}}(x_{ijk} - \bar{x}_{...})^2} \tag{47}$$

$$EV_j = \frac{\sum_{i=1}^{a} \sum_{j=1}^{b} n_{ij}(\bar{x}_{.j.} - \bar{x}_{...})^2}{\sum_{i=1}^{a} \sum_{j=1}^{b} \sum_{k=1}^{n_{ij}}(x_{ijk} - \bar{x}_{...})^2} \tag{48}$$

where $\bar{x}$ is the average of x_{ijk} over the indices indicated by the dots.

1.4.3 Mutual Information (MI)

The MI between a discriminant axis $\boldsymbol{u}$ and a feature quantifies how much information of the feature is obtained by observing data projected along that axis. It is calculated as the MI between data representations along the axis $\boldsymbol{y} = \boldsymbol{u}^T\boldsymbol{X}$ and feature labels of the data $\boldsymbol{f}$, where $\boldsymbol{X}$ is the original gene expression matrix:

$$\begin{aligned} I(\boldsymbol{y}, \boldsymbol{f}) &= H(\boldsymbol{y}) + H(\boldsymbol{f}) - H(\boldsymbol{y}, \boldsymbol{f}) \\ &= -\sum_{y \in Y} p(y) \log_2 p(y) - \sum_{f \in F} p(f) \log_2 p(f) - \sum_{y \in Y} \sum_{f \in F} p(y, f) \log_2 p(y, f) \end{aligned} \tag{49}$$

Here H indicates entropy. To calculate $H(\boldsymbol{y})$ and $H(\boldsymbol{y}, \boldsymbol{f})$, we discretize $\boldsymbol{y}$ into 10 bins.

1.4.4 Modularity

Ridgeway and Mozer (2018) argued that in a modular representation, each axis should depend on at most a single feature [2]. Following the arguments in their paper, the modularity score is computed as follows: we first calculate the MI between each feature and each axis (m_{if} denotes the MI between

one axis i and one feature f). If an axis is perfectly modular, it will have high mutual information for only one feature and zeros for the others, we therefore compute a template t_{if} as the following:

$$t_{if} = \begin{cases} \theta_i, & \text{if } f = \arg\max_g m_{ig} \\ 0, & \text{otherwise} \end{cases} \tag{50}$$

where $\theta_i = \max_g m_{ig}$. We then calculate the deviation from the template as:

$$\delta_i = \frac{\sum_f (m_{if} - t_{if})^2}{\theta_i^2 (N-1)} \tag{51}$$

where N is the number of features. The modularity score for the axis i is $1 - \delta_i$. The mean of $1 - \delta_i$ over i is defined as the overall modularity score.

1.4.5 Silhouette Score

The Silhouette score can be used to measure separation between clusters. The formula for the Silhouette score of one sample point is given by:

$$s(i) = \frac{b(i) - a(i)}{\max\{a(i), b(i)\}} \tag{52}$$

where: $a(i)$ is the average distance from the ith sample to the other samples in the same cluster, and $b(i)$ is the smallest average distance from the ith sample to the samples in the other clusters.

The Silhouette score ranges from -1 to 1. If the score is close to 1, it means that the sample is appropriately clustered. If the score is close to -1, then by the same logic, one could tell that it would be more appropriate if the data was clustered in its neighbouring cluster. The overall Silhouette score is the average silhouette score of all samples, serving as a measure of how appropriately the data have been separated and clusters.

1.5 E. Implementation details of annotation perturbation

We conducted an annotation perturbation analysis to assess the impact of incorrect phenotypic labeling on the performance of our FLDA method. For this, we utilized the T4/T5 neurons dataset and introduced three distinct types of perturbations to the original labels:

Firstly, we interchanged the phenotype labels of T4a neurons with one of the seven remaining types (T4b, T4c, T4d, T5a, T5b, T5c, T5d). In this case, phenotype labels for two cell types were erroneous, while the total number of cell type clusters remained the same. We maintained two levels for dendritic phenotypes (T4/T5) and four levels for axonal phenotypes (a/b/c/d). As per our methodological setup, we used one dimension to represent the dendritic feature and three dimensions for the axonal feature.

Secondly, we combined the axonal phenotypic level a with another level (b/c/d) to create an inaccurate new level (a+b/a+c/a+d). Under this scenario, there were three axonal phenotypes, we employed two dimensions to represent the axonal feature.

Lastly, we arbitrarily divided each of the four axonal lamination labels (a/b/c/d) into two distinct levels. For example, among neurons with the original axonal level a, we designated some of them with a level a1, while the rest were assigned a level a2. This resulted in eight axonal phenotypes (a1/a2/b1/b2/c1/c2/d1/d2), and following our methodology, we designated seven dimensions to account for the axonal feature.

In each scenario, we applied FLDA on the T4/T5 neurons dataset using the altered annotations and measured the respective metrics. These metrics were subsequently compared with those derived from the original annotation to assess the robustness of our method to mislabeling.

References

[1] Ronald Aylmer Fisher. *The Correlation between Relatives on the Supposition of Mendelian Inheritance*. Royal Society of Edinburgh], 1918.

[2] Karl Ridgeway and Michael C. Mozer. Learning deep disentangled embeddings with the F-statistic loss. In *Proceedings of the 32nd International Conference on Neural Information Processing Systems*, NIPS'18, pages 185–194, Red Hook, NY, USA, December 2018. Curran Associates Inc.
